# Cardiac manifestations in SARS-CoV-2-associated multisystem inflammatory syndrome in children: a comprehensive review and proposed clinical approach

**DOI:** 10.1007/s00431-020-03766-6

**Published:** 2020-08-15

**Authors:** Francesca Sperotto, Kevin G. Friedman, Mary Beth F. Son, Christina J. VanderPluym, Jane W. Newburger, Audrey Dionne

**Affiliations:** 1 Department of Cardiology, Boston Children’s Hospital, 300 Longwood Avenue, Boston, MA 02115 USA; 2 Department of Pediatrics, Harvard Medical School, 300 Longwood Avenue, Boston, MA 02115 USA; 3 Department of Women’s and Children’s Health, University of Padova, Via Giustiniani 2, Padua, Italy; 4 Division of Immunology, Boston Children’s Hospital, 300 Longwood Avenue, Boston, MA 02115 USA

**Keywords:** Multisystem inflammatory syndrome in children, COVID-19, SARS-CoV-2, Cardiac involvement, Myocardial dysfunction, Coronary aneurysm

## Abstract

Initial reports on COVID-19 described children as largely spared from severe manifestations, with only 2–6% of children requiring intensive care treatment. However, since mid-April 2020, clusters of pediatric cases of severe systemic hyperinflammation and shock epidemiologically linked with COVID-19 have been reported. This condition was named as SARS-Cov-2-associated multisystem inflammatory syndrome in children and showed similarities to Kawasaki disease. Here, we present a narrative review of cases reported in literature and we discuss the clinical acute and follow-up management of these patients. Patients with SARS-Cov-2-associated multisystem inflammatory syndrome frequently presented with persistent fever, gastrointestinal symptoms, polymorphic rash, conjunctivitis, and mucosal changes. Elevated inflammatory markers and evidence of cytokine storm were frequently observed. A subset of these patients also presented with hypotension and shock (20–100%) from either acute myocardial dysfunction or systemic hyperinflammation/vasodilation. Coronary artery dilation or aneurysms have been described in 6–24%, and arrhythmias in 7–60%. Cardiac support, immunomodulation, and anticoagulation are the key aspects for the management of the acute phase. Long-term structured follow-up of these patients is required due to the unclear prognosis and risk of progression of cardiac manifestations.

*Conclusion*: Multisystem inflammatory syndrome is a novel syndrome related to SARS-CoV-2 infection. Evidence is still scarce but rapidly emerging in the literature. Cardiac manifestations are frequent, including myocardial and coronary involvement, and need to be carefully identified and monitored over time.

**Table:** 

**What is Known:** • *Multisystem inflammatory syndrome in children (MIS-C) has been described associated with SARS-CoV-2.* **What is New:** • *Patients with MIS-C often present with fever, gastrointestinal symptoms, and shock.* • *Cardiac involvement is found in a high proportion of these patients, including ventricular dysfunction, coronary artery dilation or aneurysm, and arrhythmias.* • *Management is based on expert consensus and includes cardiac support, immunomodulatory agents, and anticoagulation.* • *Long-term follow-up is required due to the unclear prognosis and risk of progression of cardiac manifestation.*

**Electronic supplementary material:**

The online version of this article (10.1007/s00431-020-03766-6) contains supplementary material, which is available to authorized users.

## Introduction

Initial reports during the early phase of the COVID-19 pandemic indicated that children were relatively spared from severe manifestations, with 2–6% of children presenting with severe illness [1–3]. However, since mid-April 2020, clusters of pediatric cases of severe systemic hyperinflammation and shock epidemiologically linked with COVID-19 were reported. Riphagen et al. first described a case series of 8 previously asymptomatic children presenting with hyperinflammatory shock, ventricular dysfunction, and multiorgan involvement [4]. This was followed by other reports of patients with Kawasaki disease (KD) and KD-like syndrome, frequently complicated by significant cardiac involvement [5–9].

The increasing number of reported cases led to a health advisory from the Royal College of Pediatrics and Child Health (RCPCH), the Centers for Disease Control and Prevention (CDC), and the World Health Organization (WHO), which identified these cases as a novel condition named multisystem inflammatory syndrome in children (MIS-C), also called pediatric multisystem inflammatory syndrome (PMIS) [10–12]. For the purpose of this review, the term MIS-C will be used. Here, we aim to review the published case reports and case series of patients with MIS-C, summarize the existing evidence on its cardiac manifestations in a form of narrative review, and propose a consensus-based approach for the management of MIS-C. Methods of the review process are reported as Supplemental material.

## Definition of MIS-C

The RCPCH’s, CDC’s, and the WHO’s definitions of the novel syndrome are shown in Table 1 [10–12]. All three definitions include presence of fever, laboratory evidence of inflammation, and multisystem organ involvement without alternative plausible diagnoses, as well as evidence of COVID-19 infection or recent exposure to a COVID-19 case. The duration of fever, criteria for organ involvement, and need for documentation of SARS-CoV-2 infection vary between definitions.

**Table 1:** Case definitions for SARS-CoV-2-associated multisystem inflammatory syndrome in children

Royal College of Paediatrics and Child Health, UK	Centers for Disease Control and Prevention (CDC), USA	World Health Organization (WHO)
A child presenting with persistent fever (> 38.5 °C), inflammation (neutrophilia, elevated CRP, and lymphopenia) and evidence of single or multiorgan dysfunction (shock, cardiac, respiratory, kidney, gastrointestinal, or neurological disorder) with additional features*. This may include children fulfilling full or partial criteria for KD. Exclusion of any other microbial cause, including bacterial sepsis, staphylococcal or streptococcal shock syndromes, infections associated with myocarditis such as enterovirus (waiting for results of these investigations should not delay seeking expert advice). SARS-CoV-2 RT-PCR test results may be positive or negative. *Additional features: Clinical: Most: oxygen requirement, hypotension Some: abdominal pain, confusion, conjunctivitis, cough, diarrhea, headache, lymphadenopathy, mucus membrane changes, neck swelling, rash, respiratory symptoms, sore throat, swollen hands and feet, syncope vomiting; Laboratory: All: abnormal fibrinogen, high D-dimers, high ferritin, hypoalbuminemia; Some: acute kidney injury, anemia, thrombocytopenia, coagulopathy, high IL-10, high IL-6, proteinuria, high CK, high LDH, high TG, high troponin, transaminitis; Imaging: Echo and ECG: myocarditis, valvulitis, pericardial effusion, coronary artery dilation; CXR: patchy symmetrical infiltrates, pleural effusion; Abdo USS: colitis, ileitis, lymphadenopathy, ascites, hepatosplenomegaly; CT chest: as for CXR. May demonstrate coronary artery abnormalities if with contrast.	An individual aged < 21 years presenting with fever*, laboratory evidence of inflammation**, and evidence of clinically severe illness requiring hospitalization, with multisystem (≥ 2) organ involvement (cardiac, renal, respiratory, hematologic, gastrointestinal, dermatologic or neurological); **AND** No alternative plausible diagnoses; **AND** Positive for current or recent SARS-CoV-2 infection by RT-PCR, serology, or antigen test, or COVID-19 exposure within 4 weeks prior to the onset of symptoms. *Fever ≤ 38 °C for ≥ 24 h, or report of subjective fever lasting ≥ 24 h. **Including, but not limited to, one or more of the following: an elevated CRP, ESR, fibrinogen, procalcitonin, d-dimer, ferritin, LDH, or IL-6, elevated neutrophils, reduced lymphocytes and low albumin. Additional comments: Some individuals may fulfill or partial criteria for KD but should reported if they meet the case definition for MIS-C; Consider MIS-C in any pediatric death with evidence of SARS-Cov-2 infection.	Children and adolescents 0–19 years of age with fever ≥ 3 days; **AND** two of the following: 1. Rash or bilateral non-purulent conjunctivitis or muco-cutaneous inflammation signs (oral, hands or feet). 2. Hypotension or shock. 3. Features of myocardial dysfunction, pericarditis, valvulitis, or coronary abnormalities (including echo findings or elevated troponin/NT-proBNP), 4. Evidence of coagulopathy (by PT, PTT, elevated d-dimers). 5. Acute gastrointestinal problems (diarrhea, vomiting, or abdominal pain). **AND** Elevated markers of inflammation such as ESR, C-reactive protein, or procalcitonin. **AND** No other obvious microbial cause of inflammation, including bacterial sepsis, staphylococcal or streptococcal shock syndromes. **AND** Evidence of COVID-19 **(**RT-PCR, antigen test or serology positive), or likely contact with patients with COVID-19. Consider this syndrome in children with features of typical or atypical KD or toxic shock syndrome

*APTT* activated partial thromboplastin time, *CK* creatine kinase, *COVID-19* coronavirus disease 2019, *CXR* chest X-ray, *CRP* C-reactive protein, *echo* echocardiography, *ESR* erythrocyte sedimentation rate, *IL* interleukin, *KD* Kawasaki disease, *LDH* lactic acid dehydrogenase, *MIS-C* multisystem inflammatory syndrome in children, *NT-proBNP* N-terminal pro–B-type natriuretic peptide, *PT* prothrombin time, *PTT* partial thromboplastin time, *RT-PCR* reverse transcriptase–polymerase chain reaction, *SARS-CoV-2* severe acute respiratory syndrome coronavirus 2, *TG* triglycerides

## Clinical presentation

### Clinical symptoms

Children with MIS-C commonly present with persistent fever, asthenia, diffuse erythematous polymorphic rash, non-purulent conjunctivitis, and prominent gastrointestinal symptoms (Table 2) [4–9, 13–29]. Other commonly reported symptoms are mucosal changes and peripheral edema, which, along with the rash and conjunctivitis, resemble the clinical characteristics of KD [5–9, 13–31]. In contrast with adults, odynophagia and respiratory symptoms were rarely seen [4, 9, 14, 15, 22–27]. Notably, a subset of patients presents with hypotension and shock from either acute myocardial involvement or systemic hyperinflammation/vasodilation, frequently requiring intensive care admission, circulatory, and respiratory support (Tables 2 and 3) [4, 5, 8, 9, 13–20, 22–25, 27].

**Table 2:** Demographic, clinical characteristics and cardiac involvement in published cases of patients presenting with possible MIS-C

Author, year	Study design, setting, and period	*N*	Age, gender	Other patients’ baseline characteristics	Symptoms	Cardiac involvement					SARS-CoV-2 test	
						Ventricular function	Coronary involvement	Arrhythmia/ECG changes	Troponin	proBNP/BNP	RT-PCR	Serology
Jones 2020	Case report, Palo Alto, USA, April 2020	1	6 months, F	Previously healthy	Persistent fever, rash, conjunctivitis, mucosal changes, peripheral edema, minimal respiratory symptoms, irritability	Normal	Normal coronary arteries	-	-	-	Pos	-
Riphagen 2020	Case series, London, UK, April 2020	8	Range 4–14 years, 5M, 3F	6/8 previously healthy, 1/8 allergic rhinitis and alopecia areata, 1/8 autism, 6/8 Afro-Caribbean	Persistent fever, 7/8 GI symptoms, 5/8 conjunctivitis, 4/8 rash, 3/8 odynophagia, 2/8 headache, 1/8 myalgia, 8/8 Hypotension/shock	6/8 (4/8 mild to severe LV dysfunction, 1/8 RV dysfunction, 1/8 BiV dysfunction)	8/8 echobright coronary vessels, 1/8 giant aneurysm	1/8 in context of refractory shock, requiring ECMO; other ECGs non-specific	↑	↑ proBNP	2/8 Pos	-
Rivera-Figueroa 2020	Case report, Jackson, USA, April 2020	1	5 years, M	Previously healthy, African-American	Persistent fever, GI symptoms, rash, conjunctivitis, mucosal changes, peripheral edema, shock	Normal	Normal coronary arteries		↑		Pos	-
Balasubramanian 2020	Case report, Chennai, India, April 2020	1	8 years, M	Previously healthy, Indian	Persistent fever, odynophagia, rash, conjunctivitis, mucosal changes, peripheral edema, mild respiratory distress, hypotension	Normal	Normal coronary arteries	-	-	-	Pos	-
Verdoni 2020	Case-control study, Bergamo, Italy, 2015–2020	10	Mean 7.5 years (SD 3.5), 7M, 3F	Previously healthy, Caucasian	Persistent fever, rash, conjunctivitis, peripheral edema, 6/10 GI symptoms, 4/10 mucosal changes, 4/10 meningeal signs, hypotension	5/10 LVEF < 50%	2/10 coronary aneurysms (> 4 mm)	-	↑ 5/9	↑ proBNP 10/10	2/10 Pos	3/10 IgM+ 8/10 IgG+
Belhadjer 2020	Case series, France and Switzerland (14 centers), March–April 2020	35	Median 10 years (range 1–16 years), 18 M, 17F	31/35 previously healthy, 3/35 asthma, 1/35 SLE, 6/35 overweight (BMI > 25)	Persistent fever, asthenia, 28/35 (80%) GI symptoms, 23/35 respiratory distress, 20/35 rash, mucosal changes, 11/35 meningeal signs, 6/35 chest pain, 35/35 hypotension/shock	35/35 LVEF < 50% (inclusion criteria), 10/35 LVEF < 30%, 31/35 global LV hypokinesis, 3/35 segmental wall hypokinesis, 1/35 Takotsubo	6/35 mild coronary dilatation (z score > 2), no aneurysms	1/35 ST elevation at onset, 1/35 ventricular arrhythmia	↑	↑ 35/35 proBNP or BNP	14/35 Pos	30/35: 28/35 IgG+ 2/35 IgM+
Licciardi 2020	Case report, Turin, Italy, April 2020	2	7 years, M, 12 years, M	Previously healthy, 1/2 PFAPA syndrome	Persistent fever, GI symptoms, rash, conjunctivitis, peripheral edema, hypotension/shock	2/2 Ventricular dysfunction	Normal coronary arteries	Normal ECG	↑ 2/2	↑ 1/2 proBNP	Neg	IgM+, IgG+
Deza Leon 2020	Case report, Detroit, USA, April 2020	1	6 years, F	Previously healthy	Fever, rash, conjunctivitis, peripheral edema odynophagia, respiratory distress, cardiogenic shock	Mildly diminished LVEF at onset, severe dysfunction requiring ECMO	Normal coronary arteries	Junctional cardiac rhythm	↑	-	Pos	-
Dolinger 2020	Case report, New York, USA, May 2020	1	14 years, M	Crohn disease	Persistent fever, GI symptoms, rash, hypotension	-	-	-	-	-	Pos	–-
Labé 2020	Case report, Argenteuil, France, May 2020	2	6 years, M 3 years, M	Previously healthy	Rash, conjunctivitis, mucosal changes;1/2 persistent fever, cervical lymphadenopathy	-	-	-	-	-	1/2 Pos	-
Rauf 2020	Case report, Kerala, India, April 2020	1	5 years, M	Previously healthy	Persistent fever, GI symptoms, pyuria, conjunctivitis, peripheral edema, hypotension	Moderate LV dysfunction (FE = 35%), LV global hypokinesia	Normal coronary arteries		↑	↑proBNP	Neg	-
Chiotos 2020	Case series, Philadelphia, USA, April–May 2020	6	Range 5–14 years, 1M, 5F	Previously healthy, 2/6 African-American, 2/6 Caucasian	Persistent fever, 5/6 GI symptoms, 2/6 rash, 2/6 conjunctivitis, 3/6 mucosal changes, 1/6 peripheral edema, 1/6 headache, 2/6 irritability, 4/6 respiratory failure, 6/6 shock	4/6 Mild-Moderate LV dysfunction	1/6 diffuse dilation right coronary artery (z score 3.15); 1/6 echobright coronaries	-	↑ 3/5	↑BNP	3/6 Pos	-
Waltuch 2020	Case series, New York, USA, April 2020	4	Range 5–13, 3M, 1F	2/4 previously healthy, 1/4 hypothyroidism, 1/4 asthma	Persistent fever, GI symptoms, 3/4 conjunctivitis, 2/4 rash, 2/4 cough, 2/4 fatigue, 1/4 myalgia, hypotension	Moderately depressed LV function	1/4, dilated coronary arteries, 1/4 slight ectasia, 1/4 mildly dilated coronary arteries	-	↑ 1/4	↑BNP	0/3 Pos	4/4 IgG+
Wolfler 2020	Case series, Milan, Italy, March–April 2020	5	Mean 84 m, range 2–168, 2M, 3F	Previously healthy	Persistent fever, GI symptoms, 3/4 rash, 1/5 conjunctivitis, 1/5 respiratory distress, 5/5 hypotension/shock	5/5 Mild-moderate heart dysfunction, 3/5 LVEF < 50%	Normal coronary arteries	ST,T waves anomalies, 1/5 atrial fibrillation	↑	↑proBNP	5/5 Pos	-
Grimaud 2020	Case series, Paris, France (4 centers), April 2020	20	Median 10 years (IQR 3–15), 10M, 10F	-	Persistent fever, GI symptoms, 10/20 rash, 6/20 conjunctivitis, 5/20 mucosal changes, 2/10 lymphadenopathy, 20/20 hypotension/shock	20/20 Cardiogenic/vasoplegic shock (inclusion criteria), LVEF 35% (IQR 25–55)	Normal coronary arteries	-	↑	↑BNP	10/20 Pos, others neg	15/20 IgG+
Toubiana 2020	Case series, Paris, France, April–May 2020	21	Median 8y (range 4–17), 9M, 12F	12/21 African ancestry	Persistent fever, GI symptoms, 16/21 rash, 17/21 conjunctivitis, 16/21 mucosal changes, 12/21 cervical lymphadenopathy, 12/21 irritability, 12/21 serous effusion, 15/21 hypotension/shock	16/21 myocarditis	5/21 moderately dilated coronary arteries (z score 2–2.5), 3/21 echobright coronaries	2/16 increased QT interval, ventricular arrhythmias or diffuse ST segment elevation	↑ 17/21	↑14/18	8/21 Pos	19/21 IgG+
Whittaker 2020	Case series, UK (8 centers), March–May 2020	58	Median 9 years (IQR 6–14), 25M, 33F	7/58 comorbid: 3/58 asthma, 1/58 neuro-disability, 1/58 epilepsy, 1/58 sickle cell trait, 1/58 alopecia; 22/58 Black 18/31 Asian	Persistent fever, GI symptoms, 30/58 rash, 26/58 conjunctivitis, 17/58 mucosal changes, 12/58 respiratory symptoms, 9/58 peripheral edema, 9/58 lymphadenopathy, 6/58 odynophagia, 5/58 confusion, 29/58 shock	18/29 LV dysfunction	8/58 coronary artery dilatation (z score > 2), 7/58 z score > 2.5, giant aneurysm 2/58	4/58: 1/58 1st-degree AV block, 1/58 intractable broad complex tachycardia, requiring ECMO; 1/58 atrial fibrillation, 1/58 2nd-degree AV block	N-↑	↑ proBNP 29/29	15/58 Pos	40/46 IgG+
Blondiaux 2000	Case series, Paris, April	4	Median 9 y (range 6–12), 1M, 3F	No history of cardiovascular disease	Persistent fever, GI symptoms, rash, conjunctivitis	4/4 transient systolic disfunction, 1/4 LVEF < 30%	Normal coronary arteries	1/4 ST depression, 1/4 T waves abnormalities	↑4/4	↑4/4	0/4 Pos	4/4 IgG+, 1/4 IgM+
Cheung 2020	Case series, New York, April–May 2020	17	Median 8 years (range 2–16) 8M, 9F	Previously healthy, 3/17 mild asthma; 6/17 Ashkenazi Jewish, 4/17 Asian	Persistent fever, 14/17 GI symptoms,12/17 rash, 11/17 conjunctivitis, 9/17 mucosal changes, 3/17 respiratory symptoms, 13/17 shock	11/17 normal-mild LV dysfunction 6/17 moderate-severe LV dysfunction	7/17 echobright coronaries, 1/17 medium-sized aneurysm (z score 5.2)	10/17 Non-specific ST/T-wave abnormalities, 1/17 attenuated QRS voltage. 3/17 dysrhythmias: premature ventricular contractions, non-sustained VT, sinus bradycardia	↑14/17	↑ proBNP 15/29	8/17 Pos	9/17 IgM/IgG +
Ramcharan 2020	Case series, Birmingham, April–May 2020	15	Median 9 (IQR 7–11), 11M, 4 F	6/15 African/Afro-Caribbean, 6/15 Asian	Persistent fever, 13/15 GI symptoms, 8/15 Kawasaki-like symptoms, 4/15 myalgia, 4/15 lethargy	8/15 Reduced LV fractional shortening, 12/15 LVEF < 55%	14/15 coronary artery abnormalities: 1 aneurysm, 6 ectasia, 7 prominent	9/15 abnormal PR interval, abnormal T waves	↑15/15	↑ proBNP 15/15	2/15 Pos	12/12 IgM/IgG/IgA+
Pouletty 2020	Case series, Paris (multicenter), April–May 2020	16	Median 10 years (IQR 5–12), 8 M, 8F	10/16 Previously healthy, 2/16 asthma, 4/16 overweight	Persistent fever, 13/16 GI symptoms, 13/16 rash, 15/16 conjunctivitis, 14/16 mucosal changes, 9/16 neurological signs, 6/16 lymphadenopathy, 2/16 respiratory symptoms, 1/16 arthritis, 1/16 anosmia 11/16 shock	7/16 Myocarditis, LVEF 35% (IQR 32–46)	3/16 coronary dilation, median z score 2.6 (IQR 1.7–3.7)	–	↑11/16	↑ proBNP 11/16	11/16 Pos	7/8 IgG+
Kaushik 2020	Case series, New York (3 centers), April–May 2020	33	Median 10 years (IQR 6–13), 20M, 13F	17/33 Previously healthy, 5/33 asthma, 4/33 overweight, 15/33 Hispanic or Latino, 13/33 black	Persistent fever, 23/33 GI symptoms, 14/33 rash, 12/33 conjunctivitis, 11/33 respiratory symptoms, 7/33 mucosal changes, 4/33 neurologic involvement 21/33 hypotension	21/32 LVEF < 50%, 4/32 LVEF < 30%	6/21 prominent coronary arteries, 2/21 coronary ectasia	-	N-↑	↑BNP, proBNP	11/33 Pos	27/33 IgM/IgG+
Greene 2020	Case report, New York, May 2020	1	11 years, F	Previously healthy	Persistent fever, GI symptoms, sore throat, rash, leg pain, malaise, shock	Decreased LV function	Normal coronary arteries	-	↑	↑	Pos	-
Dufort 2020	Case series, New York (multicenter), March–May 2020	99	31/99 0–5 years, 42/99 6–12 years, 26/99 13–20 years; 53M, 46F	36/95 pre-existing condition, 29 of them obesity; 31/78 black, 31/78 Hispanic, 29/78 white	Persistent fever or chills, 79/99 GI symptoms, 59/99 rash, 60/99 mucosal change, 40/99 lower respiratory symptoms, 30/99 neurologic symptoms, 27/99 upper respiratory, 11/99 chest pain, 61/99 hypotension, 10/99 shock	51/99 some degree of ventricular dysfunction, 52/99 myocarditis	9/99 coronary artery aneurysm (4/99 z score > 2.5)	-	↑ 63/89	↑ 74/82	50/94 Pos	76/77 IgG+, 3/77 IgM+
Feldstein 2020	Case-series, United States (multicenter), March–May 2020	186	Median 8 years (IQR 3–12), 115M, 71F	135 previously healthy, 51/186 at least one underlying condition excluding obesity (resp 33/186, cardiac 5/186, immunocompromising or autoimmune 10/186, 45/153 BMI-based obesity; 46/186 Black, 57/186 Hispanic or Latino)	Persistent fever, 171/186 GI symptoms, 110/186 rash, 103/55 conjunctivitis, 78/186 oral mucosal changes, 37/186 peripheral edema, 18/186 lymphadenopathy, 131/186 respiratory symptoms, 149/186 cardiovascular symptoms	90/186 Myocardial dysfunction	15/170 Coronary artery aneurysm (z score ≥ 2.5)	12/186 Arrhythmia	↑ 50/128	↑73/153	73/186 Pos	85/186 IgM/IgG+

*AV* atrio-ventricular, *BiV* biventricular, *ECG* electrocardiogram, *ECMO* extracorporeal membrane oxygenation, *F* female, *GI* gastrointestinal, *IQR* interquartile range, *LV* left ventricle, *LVEF* left ventricular ejection fraction, *M* male, *RV* right ventricle, *SARS-CoV-2* severe acute respiratory syndrome coronavirus 2, *SD* standard deviation, *VT* ventricular tachycardia

**Table 3:** Cardiac support, anti-inflammatory, antiplatelet/anticoagulation treatments, and outcomes in published cases of patients presenting with possible MIS-C

Author, year	Cardiac support treatment		Anti-inflammatory/immunomodulatory treatments			Antiplatelet/anticoagulation treatment		Outcomes	
	Inotropes/vasopressors	ECMO	IVIG	Steroids	Biologic drugs	Aspirin	Heparin	At discharge	Follow-up
Jones 2020	No	No	2 g/kg	No	No	20 mg/kg ×4/day, then 3 mg/kg/day	No	Complete recovery	Planned at 2 weeks after discharge
Riphagen 2020	8/8	1/8	8/8	5/8	1/8 infliximab	6/8 50 mg/kg	1/8	Complete recovery, ICU after 4–6 days	1/8 developed 1 giant coronary aneurysm 1 week after discharge, 1/8 died for large cerebrovascular infarct; ongoing surveillance for coronary arteries
Rivera-Figueroa 2020	No	No	1.8 g/kg	Premed only	No	40 mg/kg/day	No	Complete recovery, discharged at day 6	Complete recovery confirmed at 2 weeks
Balasubramanian 2020	No	No	2 g/kg	No	Tocilizumab 8 mg/kg	75 mg ×1/day	No	Complete recovery	-
Verdoni 2020	2/10	No	10/10 2 g/kg	8/10 MTPD 2 mg/kg/day × 5	-	10/10 3/10 50–80 mg/kg/day, 7/10 30/mg/day; then 3–5 mg/kg/day	No	Complete recovery	Planned at 8 weeks, including echocardiogram
Belhadjer 2020	28/35	10/35	25/35 (1/25 s dose)	12/35	3/35 anakinra	28/35	23/35 Therapeutic dosage	Recovery, 7/35 residual LV dysfunction	5/35 residual mild-moderate LV systolic dysfunction at last follow-up (median 12 days)
Licciardi 2020	1/2	No	1/2 2 g/kg	2/2 MTPD 2/mg/kg/die	No	-	No	Recovery	-
Deza Leon 2020	1/1	1/1	2 g/kg	No	No	1/1	1/1 on ECMO	ECMO decannulation at day 6, organs recovery day 12	-
Dolinger 2020	No	No	No	No	Infliximab 10 mg/kg	No	No	Complete recovery	Complete recovery confirmed at 2 weeks
Labé 2020	No	No	2 g/kg	No	No	-	No	1/2 discharged at day 14	-
Rauf 2020	1/1	No	2 g/kg	MTPD 30/mg/kg/day ×3	No	1/1	No	Complete recovery	-
Chiotos 2020	5/6	No	4/6 2 g/kg, 2/6 s dose 2 g/kg	5/6 MTPD 2 mg/kg/day, 2/6 MTPD 30/mg/kg/day ×3	1/10 anakinra 4/mg/kg/day	3/6 “low dose”	No	5/6 complete recovery discharged at day 8–17, 1/6 still in PICU at moment of submission	-
Waltuch 2020	3/4	No	3/4	No	2/4 tocilizumab, 1/4 tocilizumab and anakinra	-	No	-	-
Wolfler 2020	4/5	No	4/5 2 g/kg	1/5	1/5 tocilizumab	-	4/5 LMWH prophylaxis	Complete recovery	-
Grimaud 2020	19/20	No	20/20 2 g/kg	2/20	1/20 tocilizumab, 1/20 anakinra	-	No	Full left ventricular function recovery	-
Toubiana 2020	15/21	No	21/21 2 g/kg, 5/21 s dose 2 g/kg	10/21 steroids 2–10 mg/kg/day	No	21/21 3–5 mg/kg/day	No	Complete recovery, discharged at day 5–17	-
Whittaker 2020	27/58	3/58	41/58	37/58	3/58 anakinra, 8/58 infliximab	-	-	1/58 died	-
Blondiaux 2000	3/4	No	4/4	3/4	-	-	-	Complete recovery, discharged at day 13–23	-
Cheung 2020	10/17	No	13/17 2–4 g/kg	14/17 MTPN 2–30 mg/kg/day, HC 2 mg/kg/day	1/17 tocilizumab	4/17	Enoxaparin 10/17 prophylaxis, 1/17 treatment	Most patients had improved LV function at day 2–18. 1/17 mildly decreased function	At day 3–18 of follow up, all patients at home, no fatalities
Ramcharan 2020	10/15		10/15, 3/15 s dose	5/15 MTPN	-	2/15 high dose, 11/15 low dose	No	All patients discharged home with normal/improving cardiac parameters	Planned at 1 week, including ECG and echocardiogram
Pouletty 2020	6/16	No	15/16, 5/16 s dose	4/16	1/1 tocilizumab, 1/1 anakinra	15/16 (7/16 30–50 mg/kg/day, 8/16 antiaggregant dose)	-	Complete recovery, all discharged at median follow-up of 14 days	-
Kaushik 2020	17/33	1/33	18/33	17/33	12/33 tocilizumab, 4/33 anakinra	8/24	28/33 therapeutic UHF/enoxaparin, 5/33 prophylactic enoxaparin	32/33 complete recovery, discharged at day 6–10, 20/21 complete LV function recovery, 1/33 died on ECMO	-
Greene 2020	1/1	No	1/1	1/1	Tocilizumab	-	Therapeutic dose enoxaparin	Complete recovery	Planned at 2 weeks
Dufort 2020	61/99	4/99	69/99	63/99	No	-	-	76/99 discharged, 21/99 still hospitalized as of May 15, 2020, 2/99 died	-
Feldstein 2020	90/186	8/186	144/186 2 g/kg, 39/186 s dose 2 g/kg	91/186	14/186 tocilizumab or siltuximab, 24/186 anakinra	-	87/186 (heparin, enoxaparin, bivalirudin, warfarin, argatroban)	130/186 discharged, 4/186 died as of May 20, 2020	-

*ECMO* extracorporeal membrane oxygenation, *HC* hydrocortisone, *IVIG* intravenous immunoglobulins, *LMWH* low molecular weight heparin, *MTPD* methylprednisolone, *Premed* premedication, *VA* veno-arterial

### Factors associated with MIS-C

Although comorbidities have been associated with more severe disease in both adults and children with severe COVID-19 [2], their role in MIS-C remains unclear. While Belhadjer, Dufort and Feldstein et al. hypothesize that overweight patients may have a higher risk to present MIS-C [24, 25, 27], patients overall were reported to be previously healthy, and only occasionally had a baseline chronic condition such as asthma or autoimmune disorders (Table 2) [4, 8, 15, 19, 20, 25, 27, 28]. Interestingly, none of the reported patients had known congenital heart disease or preexisting cardiovascular disease. Finally, several case series have described a high proportion of African ethnicity or ancestry [4, 18, 20, 24, 25], as well as Hispanic subjects [23–25]. Future studies may help better understand the role of genetic and socioeconomic status in the pathophysiology of MIS-C.

### Evidence of SARS-CoV-2 infection

While a small number of MIS-C patients have positive SARS-CoV-2 reverse-transcriptase protein chain reaction (RT-PCR) (Table 3), the majority have either known family exposures or serologic evidence of prior infection. Time from infection to onset of MIS-C symptoms varies among studies, from a few days to months [17, 18, 25, 27]. Overall, a variable percentages of subjects, from 0 [15, 30] to 100% [16] had positive RT-PCR; however, in most of the reports, SARS-CoV-2 positivity varies between 20 and 53% (Table 2) [4, 5, 14, 17, 18, 20, 22–25, 27]. Generally, a higher percentage (75–100%) had evidence of IgG antibodies (Table 2) [5, 15, 17, 18, 20–25, 27, 30] and suggest that a postinfectious immune response may be responsible for this condition [32].

### Laboratory findings

Elevated inflammatory markers and evidence of hyperinflammation were widely reported and consistently found in patients with MIS-C [4–9, 13–33]. Supplemental Table 1 summarizes the main laboratory characteristics of the existing cases in the literature. Overall, C-reactive protein (CRP), procalcitonin (PCT), and erythrocyte sedimentation rate (ESR) are highly elevated, as well as ferritin and IL-6. A significant increase in D-dimer and fibrinogen are key features of the coagulation profile, while the hematologic aspect of the disease is characterized by leukocytosis, neutrophilia with immature forms, lymphopenia, normal or decreased red blood cell count and normal or decreased platelet count.

### Cardiac involvement

#### Myocardial dysfunction

Left ventricular (LV) systolic dysfunction has been described in a large proportion of children diagnosed with MIS-C in both the initial reports and subsequent case series. Cardiac findings in children with MIS-C are summarized in Table 2 and Table 3. In the first MIS-C case-series reported from the UK, cardiac dysfunction was present in 6/8 patients (75%) [4]. In subsequent case series, ventricular dysfunction has been reported in 35–100% of children with MIS-C, depending on definition and inclusion criteria (Table 2) [4, 5, 8, 9, 13–25, 27, 30, 31].

Two of the published case series described selected cohorts of patients with myocardial dysfunction as inclusion criteria [17, 27]. Belhadjer et al. reported a selected cohort of 35 MIS-C patients who developed acute LV failure (LV ejection fraction (LVEF) < 50%) or shock, fever, and elevated inflammatory markers [27]. Management of these patients included mechanical ventilation and inotropic support in 80% of patients, and extracorporeal membrane oxygenation (ECMO) support in 28%. All patients successfully weaned off ECMO and none had died at the time of publication. [27]. Grimaud at el. reported 20 patients admitted with cardiogenic/vasoplegic shock and a median LVEF of 35% (IQR 25–55%). Nineteen out of 20 patients required inotropes/vasopressors but no ECMO support was needed. All patients had a full recovery of the LV function prior to discharge [17].

A high proportion of patients also had elevated troponin level or B-type natriuretic peptide (BNP)/pro-BNP values (Table 2), which may be a useful marker for myocardial involvement. Most patients had recovery of ventricular function, but 6–14% of patients had persistent dysfunction at discharge (Table 3).

The mechanism underlying myocardial dysfunction in MIS-C has not been yet fully elucidated. Possible causes of myocardial injury in adults with COVID-19 include acute myocarditis, hypoxic injury, ischemic injury caused by cardiac microvascular damage or coronary artery disease, right heart strain (acute cor pulmonale), stress cardiomyopathy (Takotsubo), and systemic inflammatory response syndrome [3, 34–37]. The variable timing and modality of presentation with ventricular dysfunction suggests that different pathophysiological mechanisms may be responsible: while the acute infection may explain the occurrence of acute myocardial damage, a second phase characterized by a post-viral immunological reaction and systemic hyperinflammation may explain the occurrence of myocardial inflammation and dysfunction in predisposed subjects. In this second phase, a combination of cardiogenic and distributive shock may be observed. Advanced cardiac imaging in patients with MIS-C and ventricular dysfunction may help us better understand the underlying mechanism of injury, and presence of long-term scar or myocardial damage.

#### Coronary involvement

Coronary artery dilation or aneurysms have been described in 6–24% of patients (Table 2) [4, 5, 14, 15, 18–25, 27]. Most cases described mild coronary artery dilation with z-scores 2–2.5. As coronary artery z-scores are based on healthy, non-febrile children, some of the findings in the acute phase may be related to coronary vasodilation in the setting of fever and inflammation. However, there have also been reports of large and giant coronary artery aneurysms [4, 20], and progression of coronary aneurysm following discharge raising concerns for coronary artery intimal disruption [4, 5, 19, 20]. The late development of coronary artery aneurysm highlights the need for ongoing follow-up of those patients.

#### Arrhythmia

Studies focusing on arrhythmic manifestations have described 7–60% of patients having rhythm abnormalities of variable severity (Table 2). The most frequently reported electrocardiogram (ECG) anomalies were non-specific and included ST segment changes, QTc prolongation, and premature atrial or ventricular beats. First- and second-degree atrioventricular blocks were reported in one series, while atrial fibrillation was described in two reports [9, 20]. However, there have also been reported cases of sustained arrhythmias leading to hemodynamic collapse and need for ECMO support [4, 20].

## Hyperinflammatory state and resemblance to Kawasaki disease

MIS-C overlaps with many features of KD [5, 20, 32]. KD is an acute pediatric vasculitis involving medium-sized vessels typically affecting children < 5 years of age [38, 39]. The etiology of KD is still unknown, but it has been considered an inflammatory syndrome likely resulting from an infectious or other environmental trigger in a genetically susceptible host. While no specific infectious trigger has been confirmed, several viruses have been implicated, including coronaviruses [40, 41]. Clinical diagnostic criteria include a persistent fever (> 5 days) and at least 4 of 5 clinical symptoms including mucocutaneous involvement, non-purulent conjunctivitis, polymorphous rash, unilateral lymphadenopathy, and palmar/plantar erythema and desquamation. An incomplete form of KD is defined by persistent fever and presence of < 4 of classical symptoms with suggestive laboratory data and/or echo findings [38, 39]. In the acute phase of the disease, about 7% of patients manifest hemodynamic instability, a condition known as KD shock syndrome (KDSS) [39, 42]. Compared with KD patients without any signs of shock, KDSS patients were more frequently female, had a larger proportion of bands, higher CRP, and lower hemoglobin and platelet counts [42].

The small case series from Bergamo, Italy, reported a 30-fold increase in the incidence of KD or KD-like illness during the height of COVID-19 outbreak in the region (uncorrected for seasonal incidence), with many patients testing positive for IgG antibody and negative RT-PCR [5]. When these cases were compared with 19 classical KD, COVID-19-associated cases were found to be older (7.5 ± 3.5 vs 3.0 ± 2.5 years), more likely to present in shock (50% vs 0%), to have more cardiac involvement (abnormal echocardiogram in 60% vs 10%), and more likely to have elevation in troponin or BNP. Similarly, Whittaker et al. compared patients meeting the MIS-C definition with classical KD or KDSS patients [20], reporting that patients with MIS-C were generally older, had higher white blood cell count, neutrophil count, CRP, fibrinogen levels and higher troponin, as well as more profound lymphopenia, anemia, and lower platelet counts.

While generally self-limited, KD can have a number of long-term sequelae, the most important of which are cardiovascular. In addition to ventricular and valvular dysfunction, patients with KD can develop persistent coronary aneurysms, occurring in 20–25% of untreated children [38, 43, 44]. Coronary dilation or aneurysms have been reported in up to 25% of MIS-C patients, suggesting a pathophysiologic similarity with KD. Even if MIS-C patients have different clinical characteristics and laboratory findings compared with classical KD, the similarity in clinical features and the development of coronary artery aneurysms in both disorders may represent a key point for the future understanding of underlying pathophysiologic mechanisms [32]. Further studies will be needed to deeply understand the pathophysiology of this disorder.

## Management

Management of patients with MIS-C is reported in Table 3. Overall, admission to the intensive care unit (ICU) for management of shock was described in 20–100% of the patients, most often for inotropic support (Table 3) [4, 5, 8, 9, 13–25, 27, 30, 31]. More rarely, patients required V-A ECMO support (0–28%) [4, 9, 20, 23–25, 27]. Most patients received immunomodulatory treatment with intravenous immunoglobulin. The use of corticosteroids was less consistent and ranged from low-dose treatment to high-dose methylprednisolone pulses [4, 5, 8, 13, 14, 16–25, 27, 30, 31]. The use of anti-inflammatory dosages of aspirin has been also occasionally reported [5, 6, 21, 22]. Not infrequently, cytokine blockers have been added as a supplemental therapy, with a preference for IL-6 inhibitors (tocilizumab), but also IL-1 or tumor-necrosis-factor (TNF)-αinhibitors (anakinra, infliximab) [4, 14–17, 19, 20, 22, 23, 25, 27, 28, 31, 33]. Antiplatelet treatment with aspirin was frequently adopted, especially in patients with KD-like clinical presentations, or in those with evidence of coronary involvement. A therapeutic or prophylactic anticoagulation approach was less frequently used, except for a few case series [16, 19, 23, 25, 27, 31].

Due to the scarce knowledge and the small number of reported cases so far, the management of patients with MIS-C has been largely based on expert opinion and extrapolated from KD treatment, adult experience with COVID-19, and other systemic inflammatory disorders in children. Here, we describe a consensus-based approach for the acute and medium-term management of children with MIS-C, as well as a follow-up algorithm, developed within our Institution. However, it is necessary to emphasize that there are currently no approved therapies for MIS-C patients, and data from higher-evidence studies may quickly lead to changes in clinical practice.

### Proposed clinical approach

A multidisciplinary team should be involved in the management of patients with MIS-C, including cardiology, rheumatology, intensive care, and infectious disease specialists. Given the lack of established treatment, possibility of harm, and limited drug supply, treatment is currently not recommended for (a) prevention or postexposure prophylaxis or (b) non-hospitalized patients.

#### Cardiac support

As described above, a high proportion of patients will present with shock and require acute resuscitation. Pediatric resuscitation guidelines should be followed [45]. In patients with suspicion or evidence of ventricular dysfunction, smaller fluid boluses (10 mg/kg) should be administered with careful reassessment for signs of fluid overload between each. Extracorporeal membrane oxygenation should be considered if medical support fails.

#### Immunomodulatory therapy

There may be a benefit of immunomodulatory therapy in patients with MIS-C, severe disease, and evidence of cytokine storm syndrome and/or those with cardiac involvement. Due to recent emergence of MIS-C, no randomized trials or comparative effectiveness studies have evaluated treatment strategies, but the benefits of immunomodulatory therapy are well established in KD [39], and they are often used for the treatment of infective myocarditis [46–48] and other systemic inflammatory diseases [49, 50]. Therefore, based on the experience in similar conditions, it appears reasonable to suggest an immunomodulation approach based on intravenous immunoglobulins (IVIGs). Slower IVIG administration should be considered in patients with myocardial dysfunction to decrease the risk of fluid overload. Low-dose corticosteroids should be considered in sicker patients, in patients with known baseline conditions which can benefit from steroid treatment, or based on clinical judgment. The use of biologic drugs (tocilizumab, anakinra, infliximab) could be considered in patients with severe or critical illness, especially if they did not respond to first-line treatments.

#### Antiplatelet treatment and anticoagulation

Children with MIS-C are at risk of thrombotic complications from multiple causes, including hypercoagulable state, possible endothelial injury, stasis from immobilization, ventricular dysfunction, and coronary artery aneurysm. For these reasons, antiplatelet and/or anticoagulation treatment is recommended. Decisions about anticoagulation should be based on coagulation tests, viscoelastic testing [51, 52], and clinical presentation. Patients with evidence of myocardial involvement or coronary artery dilation may benefit from antiplatelet therapy and prophylactic anticoagulation. In addition, therapeutic anticoagulation may be considered in patients with very abnormal coagulation profile (i.e., D-dimer ≥ 3 mg/mL), documented thrombosis, arrhythmia, ventricular dysfunction greater than moderate, or giant coronary artery aneurysm. However, it should be emphasized that this is based on experts’ opinion, with no evidence to support recommendations.

#### Antiviral therapy

The role of antiviral therapies (e.g., remdesivir) in the management of children with MIS-C is uncertain [53, 54]. Evidence suggests that MIS-C represents a postinfectious complication rather than an active infection. Although we did not include antiviral therapies as an established step in our algorithm, antiviral therapy may be considered in patients with severe manifestations and concerns for ongoing infection with positive RT-PCR. A consultation with specialists in infectious disease is highly recommended in this case.

#### Outpatient follow-up

Cardiac manifestations often improve and/or normalize prior to hospital discharge, but some patients have shown residual cardiac lesions. Additionally, some series reported progression of coronary artery aneurysm following discharge, highlighting our limited knowledge of this disease and the potential for long-term complications. Therefore, it is essential to guarantee an adequate medium and long term follow-up to these patients.

**Fig. 1:**
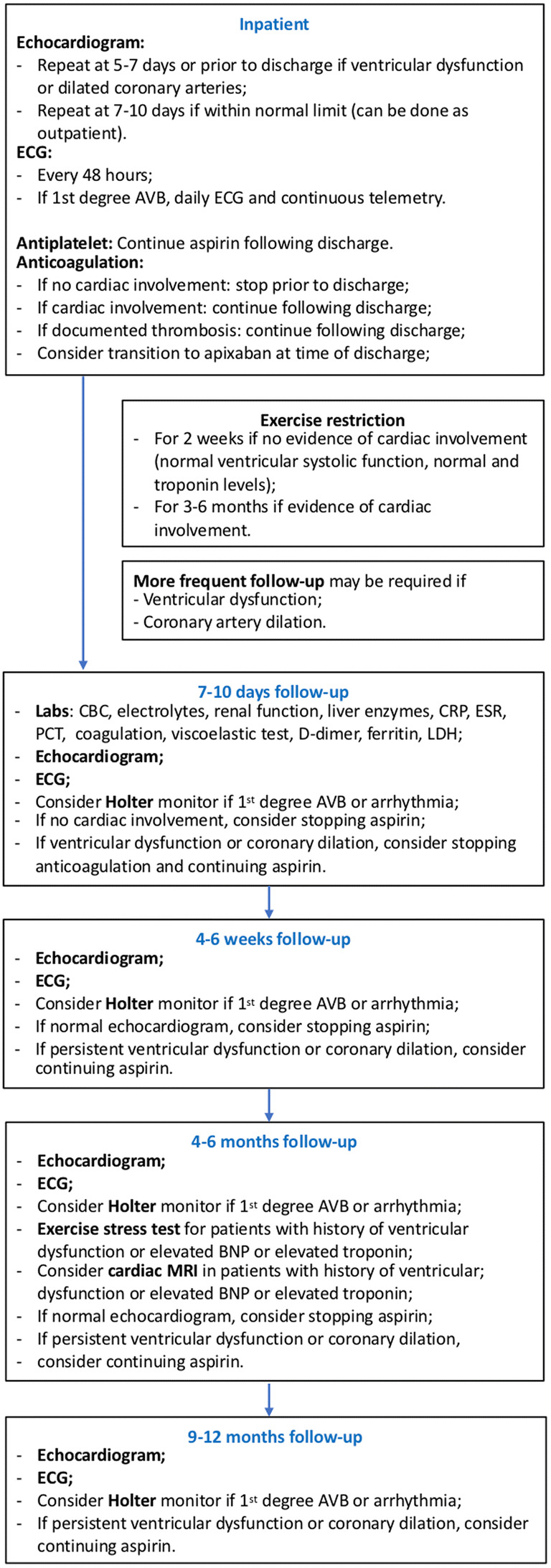
Suggested outpatient follow-up of patients with MIS-C. AVB atrioventricular block, ECG electrocardiogram, CBC complete blood count, CRP C-reactive protein, ESR erythrocyte sedimentation rate, MRI magnetic resonance imaging, PCT procalcitonin, LDH lactic dehydrogenase

At this point of knowledge, we recommend follow-up for at least a year after initial diagnosis (Fig. 1). At initial visits, laboratory testing should be obtained to document normalization of inflammatory markers and resolution of hematologic anomalies. Laboratory testing may also guide weaning of corticosteroids if used in the acute phase. Echocardiograms should be obtained at regular intervals for evaluation of ventricular function and coronary artery dimensions. ECGs should also be obtained due to reports of arrhythmias including atrioventricular block, which may progress after initial diagnosis. If anomalies are identified on ECG, Holter monitors may be useful as further investigation. In patients with a history of ventricular dysfunction, cardiac magnetic resonance imaging (MRI) may be considered 2–6 months after initial diagnosis for evaluation of ventricular function, edema, diffuse fibrosis, and scar.

While the prothrombotic risk is greatest in the acute phase, the optimal duration of antiplatelet and anticoagulation remains unclear. Patients with documented thrombosis should be continued on anticoagulation for at least 3 months after discharge. Coagulation, D-dimer, and viscoelastic testing may help guide discontinuation of anticoagulation. Moreover, patients with persistent ventricular dysfunction and/or coronary artery aneurysm may also benefit from long-term antiplatelet and/or anticoagulation depending on the severity of cardiac involvement.

#### Exercise restriction

Due to the high prevalence of myocardial involvement with MIS-C, the safety to return to physical activity and exercise after discharge is unanswered. While the etiology of the myocardial involvement remains unknown, it is clear that there are similarities to acute myocarditis. Thus, one can argue that guidelines for return to sport participation after myocarditis should be followed in those patients [48, 55]. After acute myocarditis, restriction from physical activity for at least 6 months following diagnosis is recommended. Preparticipation evaluation with echocardiograms and exercise testing may be beneficial to document the safety of exercise participation.

## Conclusion

MIS-C is a novel syndrome related to SARS-CoV-2 infection characterized by fever, signs of inflammation, and organ dysfunction. Evidence is still scarce but rapidly emerging from literature. Myocardial involvement, due to either acute myocarditis or secondary hyperinflammation, is frequent in children with MIS-C. Coronary dilation or aneurysm and arrhythmias may develop and evolve over time. Cardiac support, immunomodulation, and antiplatelet/anticoagulation treatments are part of the management of acute MIS-C. Finally, follow-up of MIS-C patients is essential to better understand the evolution and prognosis of this disease. Future studies are needed to define evidence-based management of this novel condition.

## Electronic supplementary material

ESM 1 (DOCX 24 kb)

## Abbreviations

BNP: B-type natriuretic peptide
BMI: Body mass index
CDC: Centers for Disease Control and Prevention
COVID-19: Coronavirus disease 2019
CRP: C-reactive protein
ECG: Electrocardiogram
ECMO: Extracorporeal membrane oxygenation
ESR: Erythrocyte sedimentation rate
ICU: Intensive care unit
IL: Interleukin
KD: Kawasaki disease
KDSS: Kawasaki disease shock syndrome
LV: Left ventricular
LVEF: LV ejection fraction
MIS-C: Multisystem inflammatory syndrome in children
MRI: Magnetic resonance imaging
PCT: Procalcitonin
RCPCH: Royal College of Pediatrics and Child Health
RT-PCR: Reverse-transcriptase protein chain reaction
SARS-CoV-2: Severe acute respiratory syndrome coronavirus 2
TNF: Tumor necrosis factor
WHO: World Health Organization

## References

[1] Dong Y, Mo X, Hu Y, Qi X, Jiang F, Jiang Z, Tong S (2020). Epidemiology of COVID-19 among children in China. Pediatrics.

[2] Liguoro I, Pilotto C, Bonanni M et al (2020) SARS-COV-2 infection in children and newborns: a systematic review. Eur J Pediatr. 10.1007/s00431-020-03684-7 10.1007/s00431-020-03684-7 PMC7234446 32424745

[3] Sanna G, Serrau G, Bassareo PP et al (2020) Children’s heart and COVID-19: up-to-date evidence in the form of a systematic review. Eur J Pediatr. 10.1007/s00431-020-03699-0 10.1007/s00431-020-03699-0 PMC7261213 32474800

[4] Riphagen S, Gomez X, Gonzalez-martinez C (2020). Hyperinflammatory shock in children during COVID-19 pandemic. Lancet.

[5] Verdoni L, Mazza A, Gervasoni A (2020). An outbreak of severe Kawasaki-like disease at the Italian epicentre of the SARS-CoV-2 epidemic: an observational cohort study. Lancet.

[6] Jones VG, Mills M, Suarez D, Hogan CA, Yeh D, Segal JB, Nguyen EL, Barsh GR, Maskatia S, Mathew R (2020). COVID-19 and Kawasaki disease : novel virus and novel case. Hosp Pediatr.

[7] Rivera-Figueroa EI, Santos R, Simpron S, Garg P (2020). Incomplete Kawasaki disease in a child with Covid-19. Indian Pediatr.

[8] Licciardi F, Pruccoli G, Denina M, Parodi E (2020) SARS-CoV-2 – induced Kawasaki-like hyperinflammatory syndrome : a novel COVID phenotype in children. Pediatrics. 10.1542/peds.2020-1711 10.1542/peds.2020-1711 32439816

[9] Deza Leon MP, Redzepi A, McGrath E et al (2020) COVID-19–associated pediatric multisystem inflammatory syndrome. J Pediatric Infect Dis Soc 1-2. 10.1002/jmv.25807 10.1093/jpids/piaa061 PMC7313914 32441749

[10] Royal College of Paediatrics and Child Health. Guidance: paediatric multisystem inflammatory syndrome temporally associated with COVID-19. https://www.rcpch.ac.uk/resources/guidance-paediatric-multisystem-inflammatory-syndrome-temporally-associated-covid-19 10.1111/jpc.16408 37114744

[11] Centers for Disease Control and Prevention. Emergency preparedness and response: health alert network. https://emergency.cdc.gov/han/2020/han00432.asp

[12] World Health Organization. Multisystem inflammatory syndrome in children and adolescents with COVID-19. Published May 15, 2020

[13] Rauf A, Vijayan A, John S et al (2020) Multisystem inflammatory syndrome with features of atypical Kawasaki disease during COVID-19 pandemic. Indian J Pediatr 2-4. 10.1007/s12098-020-03357-1 10.1007/s12098-020-03357-1 PMC8823324 32462354

[14] Chiotos K, Bassiri H, Behrens EM et al (2020) Multisystem inflammatory syndrome in children during the COVID-19 pandemic: a case series. J Pediatric Infect Dis Soc. 10.1093/jpids/piaa069 10.1093/jpids/piaa069 PMC7313950 32463092

[15] Waltuch T, Gill P, Zinns LE, Whitney R, Tokarski J, Tsung JW, Sanders JE (2020) Features of COVID-19 post-infectious cytokine release syndrome in children presenting to the emergency department. Am J Emerg Med. 10.1016/j.ajem.2020.05.058 10.1016/j.ajem.2020.05.058 PMC7255141 32471782

[16] Wolfler A, Mannarino S, Giacomet V (2020). Acute myocardial injury: a novel clinical pattern in children with COVID-19. Lancet child Adolesc Heal.

[17] Grimaud M, Starck J, Levy M (2020). Acute myocarditis and multisystem inflammatory emerging disease following SARS-CoV-2 infection in critically ill children. Ann Intensive Care.

[18] Toubiana J, Poirault C, Corsia A et al (2020) Kawasaki-like multisystem inflammatory syndrome in children during the covid-19 pandemic in Paris, France: prospective observational study. Br Med J 1-7. 10.1136/bmj.m2094 10.1136/bmj.m2094 PMC7500538 32493739

[19] Cheung EW, Zachariah P, Gorelik M et al (2020) Multisystem inflammatory syndrome related to COVID-19 in previously healthy children and adolescents in New York city. JAMA J Am Med Assoc 8–10. 10.1001/jama.2020.10374 10.1001/jama.2020.10374 PMC7281352 32511676

[20] Whittaker E, Bamford A, Kenny J et al (2020) Clinical characteristics of 58 children with a pediatric inflammatory multisystem syndrome temporally associated with SARS-CoV-2. JAMA - J Am Med Assoc 1-11. 10.1001/jama.2020.10369 10.1001/jama.2020.10369 PMC7281356 32511692

[21] Ramcharan T, Nolan O, Lai CY et al (2020) Paediatric inflammatory multisystem syndrome: temporally associated with SARS-CoV-2 (PIMS-TS): cardiac features, management and short-term outcomes at a UK tertiary paediatric hospital. Pediatr Cardiol 2. 10.1007/s00246-020-02391-2 10.1007/s00246-020-02391-2 PMC7289638 32529358

[22] Pouletty M, Borocco C, Ouldali N et al (2020) Paediatric multisystem inflammatory syndrome temporally associated with SARS-CoV-2 mimicking Kawasaki disease (Kawa-COVID-19): a multicentre cohort. Ann Rheum Dis 999–1006. 10.1136/annrheumdis-2020-217960 10.1136/annrheumdis-2020-217960 PMC7299653 32527868

[23] Kaushik S, Aydin SI, Derespina KR et al (2020) Multisystem inflammatory syndrome in children (MIS-C) associated with SARS-CoV-2 infection: a multi-institutional study from New York City. J Pediatr 2-7. 10.1016/j.jpeds.2020.06.045 10.1016/j.jpeds.2020.06.045 PMC7293760 32553861

[24] Dufort EM, Koumans EH, Chow EJ et al (2020) Multisystem inflammatory syndrome in children in New York state. N Engl J Med 1-12. 10.1056/NEJMoa2021756 10.1056/NEJMoa2021756 PMC7346766 32598830

[25] Feldstein LR, Rose EB, Horwitz SM et al (2020) Multisystem inflammatory syndrome in U.S. children and adolescents. N Engl J Med 1-13. 10.1056/NEJMoa2021680 10.1056/NEJMoa2021680 PMC7346765 32598831

[26] Balasubramanian SK, Tiruvoipati R, Amin M, Aabideen KK, Peek GJ, Sosnowski AW, Firmin RK (2007). Factors influencing the outcome of paediatric cardiac surgical patients during extracorporeal circulatory support. J Cardiothorac Surg.

[27] Belhadjer Z, Méot M, Bajolle F et al (2020) Acute heart failure in multisystem inflammatory syndrome in children (MIS-C) in the context of global SARS-CoV-2 pandemic. Circulation 33. 10.1161/CIRCULATIONAHA.120.048360 10.1161/CIRCULATIONAHA.120.048360 32418446

[28] Dolinger MT, Person H, Smith R et al (2019) Pediatric Crohn’s disease and multisystem inflammatory syndrome in children (MIS-C) and COVID-19 treated with infliximab. J Pediatr Gastroenterol Nutr. 10.1097/MPG.0000000000002809 10.1097/MPG.0000000000002809 PMC7268863 32452979

[29] Labé P, Ly A, Sin C et al (1827) Erythema multiforme and Kawasaki disease associated with COVID-19 infection in children. J Eur Acad Dermatol Venereol 0-1. 10.1111/jdv.16666 10.1111/jdv.16666 PMC7283825 32455505

[30] Blondiaux E, Parison P, Redheuil A (2012). Cardiac MRI of children with multisystem inflammatory syndrome (MIS-C) associated with COVID-19: case series Eléonore. Radiology.

[31] Greene AG, Saleh M, Roseman E, Sinert R (2020). Toxic shock-like syndrome and COVID-19: a case report of multisystem inflammatory syndrome in children (MIS-C). Am J Emerg Med.

[32] Mccrindle BW, Manlhiot C (2020) SARS-CoV-2-related inflammatory multisystem syndrome in children: different or shared etiology and pathophysiology as Kawasaki disease? JAMA - J Am Med Assoc 8–10. 10.1038/ng.2007.59 10.1001/jama.2020.10370 32511667

[33] Balasubramanian S, Nagendran T, Ramachandran B, Ramanan A (2020) Hyper-inflammatory syndrome in a child With COVID-19 treated successfully with intravenous immunoglobulin and tocilizumab. Indian Pediatr 1–5. 10.1007/s13312-020-1901-z 10.1007/s13312-020-1901-z PMC7387261 32393681

[34] Shi S, Qin M, Shen B et al (2020) Association of cardiac injury with mortality in hospitalized patients with COVID-19 in Wuhan, China. JAMA Cardiol 1-8. 10.1001/jamacardio.2020.0950 10.1001/jamacardio.2020.0950 PMC7097841 32211816

[35] Creel-Bulos C, Hockstein M, Amin N et al (2020) Acute cor pulmonale in critically ill patients. N Engl J Med 89-92. 10.1056/NEJMc2010459 10.1056/NEJMc2010459 PMC7281714 32374956

[36] Zheng YY, Ma YT, Zhang JY, Xie X (2020). COVID-19 and the cardiovascular system. Nat Rev Cardiol.

[37] Tersalvi G, Vicenzi M, Calabretta D, Biasco L, Pedrazzini G, Winterton D (2020). Elevated troponin in patients with coronavirus disease 2019: possible mechanisms. J Card Fail.

[38] Son MBF, Newburger JW (2018). Kawasaki disease. Pediatr Rev.

[39] McCrindle BW, Rowley AH, Newburger JW (2017). Diagnosis, treatment, and long-term management of Kawasaki disease. Circulation.

[40] Esper F, Shapiro ED, Weibel C, Ferguson D, Landry ML, Kahn JS (2005). Association between a novel human coronavirus and Kawasaki disease. J Infect Dis.

[41] Turnier JL, Anderson MS, Heizer HR, Jone PN, Glode MP, Dominguez SR (2020). Concurrent respiratory viruses and Kawasaki disease. Pediatrics.

[42] Kanegaye JT, Wilder MS, Molkara D, Frazer JR, Pancheri J, Tremoulet AH, Watson VE, Best BM, Burns JC (2009). Recognition of a Kawasaki disease shock syndrome. Pediatrics.

[43] Friedman KG, Gauvreau K, Hamaoka-okamoto A (2016). Coronary artery aneurysms in Kawasaki disease : risk factors for progressive disease and adverse cardiac events in the US population. J Am Hear Assoc.

[44] Liu L, Luo C, Hua Y, Wu M, Shao S, Liu X, Zhou K, Wang C (2020). Risk factors associated with progression and persistence of small- and medium-sized coronary artery aneurysms in Kawasaki disease: a prospective cohort study. Eur J Pediatr.

[45] Edelson DP, Sasson C, Chan PS et al (2020) Interim guidance for basic and advanced life support in adults, children, and neonates with suspected or confirmed COVID-19: from the Emergency Cardiovascular Care Committee and Get With the Guidelines-Resuscitation Adult and Pediatric Task Forces of the American Heart Association. Circulation 1–12. 10.1161/CIRCULATIONAHA.120.047463 10.1161/CIRCULATIONAHA.120.047463 PMC7302067 32270695

[46] Drucker NA, Colan SD, Lewis AB, Beiser AS, Wessel DL, Takahashi M, Baker AL, Perez-Atayde AR, Newburger JW (1994). Y-globulin treatment of acute myocarditis in the pediatric population. Circulation.

[47] Heidendael JF, Den Boer SL, Wildenbeest JG et al (2020) Intravenous immunoglobulins in children with new onset dilated cardiomyopathy. Cardiol Young 46–54. 10.1017/S1047951117001561 10.1017/S1047951117001561 28797313

[48] Canter CE, Simpson KE (2014). Diagnosis and treatment of myocarditis in children in the current era. Circulation.

[49] Bayry J, Negi VS, Kaveri SV (2011). Intravenous immunoglobulin therapy in rheumatic diseases. Nat Rev Rheumatol.

[50] de Chambrun MP, Luyt C-E, Beloncle F (2017). The clinical picture of severe systemic capillary-leak syndrome episodes requiring ICU admission. Crit Care Med.

[51] Wright FL, Vogler TO, Moore EE et al (2020) Fibrinolysis shutdown correlates to thromboembolic events in severe COVID-19 infection. J Am Coll Surg:1–11. 10.1016/j.jamcollsurg.2020.05.007 10.1016/j.jamcollsurg.2020.05.007 PMC7227511 32422349

[52] Raval JS, Burnett AE, Rollins-Raval MA et al (2020) Viscoelastic testing in COVID-19: a possible screening tool for severe disease? Transfusion 4-5. 10.1111/trf.15847 10.1111/trf.15847 PMC7267656 32374920

[53] Beigel JH, Tomashek KM, Dodd LE et al (2020) Remdesivir for the treatment of Covid-19 - preliminary report. N Engl J Med 1-12. 10.1056/NEJMoa2007764 10.1056/NEJMc2022236 32649078

[54] Goldman JD, Lye DCB, Hui DS et al (2020) Remdesivir for 5 or 10 days in patients with severe Covid-19. N Engl J Med 1-11. 10.1056/NEJMoa2015301 10.1056/NEJMoa2015301 PMC7377062 32459919

[55] Caforio ALP, Pankuweit S, Arbustini E, Basso C, Gimeno-Blanes J, Felix SB, Fu M, Heliö T, Heymans S, Jahns R, Klingel K, Linhart A, Maisch B, McKenna W, Mogensen J, Pinto YM, Ristic A, Schultheiss HP, Seggewiss H, Tavazzi L, Thiene G, Yilmaz A, Charron P, Elliott PM, European Society of Cardiology Working Group on Myocardial and Pericardial Diseases (2013). Current state of knowledge on aetiology, diagnosis, management, and therapy of myocarditis: a position statement of the European Society of Cardiology Working Group on Myocardial and Pericardial Diseases. Eur Heart J.

