# Supplementary material for: Cardiac manifestations in SARS-CoV-2-associated multisystem inflammatory syndrome in children: a comprehensive review and proposed clinical approach
Source: Eur J Pediatr. 2020 Aug 15;180(2):307–22. doi: 10.1007/s00431-020-03766-6 (PMC7429125; doi:10.1007/s00431-020-03766-6)
Supplement: Supplementary file 1 — (DOCX 24 kb) [file 431_2020_3766_MOESM1_ESM.docx]

**Cardiac manifestations in SARS-CoV-2-associated Multisystem Inflammatory Syndrome in Children:**

A comprehensive narrative review and proposed clinical approach

Francesca Sperotto, Kevin G. Friedman, Mary Beth F. Son, Christina J. Vanderpluym,

Jane W. Newburger, Audrey Dionne

**Supplemental material**

**Content details:**

- **Supplemental Table 1:** Laboratory findings in published cases of patients presenting with possible MIS-C
- **Supplemental Methods:** Data source, search strategy and study selection

**Supplemental Table 1. Laboratory findings in published cases of patients presenting with possible MIS-C**

| **Author, year** | **Laboratory findings** | | | | | | | | | | |
| --- | --- | --- | --- | --- | --- | --- | --- | --- | --- | --- | --- |
|  | **WBC** | **RBC** | **Plts** | **CRP** | **ESR** | **PCT** | **Ferritin** | **D-dimer** | **Albumin** | **IL-6** | **Others** |
| Jones  2020 | Left-shifted | ↓ | N | ↑ | ↑ | - | - | - | ↓ | - | HypoNa |
| Riphagen  2020 | - | - | ↓ | ↑ | ↑ | ↑ | ↑ | ↑ | ↓ | - | - |
| Rivera-Figueroa  2020 | ↑ | ↓ | ↓ | ↑ | ↑ | ↑ | ↑ | - | ↓ | - | HypoNa,  ↑ liver enzymes |
| Balasubramanian  2020 | ↑  Neutrophilia | ↓ | N | ↑ | ↑ | - | ↑ | - | ↓ | - | HypoNa |
| Verdoni  2020 | ↑  Neutrophilia, lymphopenia | ↓-N | ↓ | ↑ | ↑ | - | ↑ | N-↑ | N | N-↑ | HypoNa, N-↑liver enzymes, ↑ TG,  ↑fibrinogen |
| Belhadjer  2020 | ↑  Neutrophilia | - | - | ↑ | - | ↑ | - | ↑ | - | ↑ | - |
| Licciardi  2020 | Lymphopenia | - | ↓ | ↑ | ↑ | ↑ | ↑ | ↑ | ↓ | - | ↑fibrinogen  ↓complement |
| Deza Leon  2020 | ↑  Neutrophilia,  bands | ↓ | N | ↑ | ↑ | - | ↑ | ↑ | ↓ | - | ↑LDH  ↑fibrinogen  hypoNa,  hyperK |
| Dolinger  2020 | - | - | - | ↑ | ↑ | - | ↑ | ↑ | ↓ | ↑ | ↑IL-8, ↑TNFα  ↑liver enzymes |
| Labé  2020 | ↑ | - | - | ↑ | - | - | - | - | - | - | - |
| Rauf  2020 | ↑  Neutrophilia | - | N | ↑ | ↑ | - | ↑ | - | ↓ | - | hypoNa,  ↑liver enzymes ↑creatinine |
| Chiotos  2020 | N-↑  lymphopenia | N-↓ | N-↓ | ↑ | ↑ | ↑ | ↑ | ↑ | N-↓ | - | HypoNa,  N-↑creatinine,  N-↑liver enzymes |
| Waltuch  2020 | N-↑  lymphopenia | N | N-↓ | ↑ | ↑ | ↑ | ↑ | ↑ | - | ↑ | ↑LDH,  N-↑liver enzymes, ↑IL-8, ↑IL-1- β, ↑TNF-alfa |
| Wolfler  2020 | N  Neutrophilia | N-↓ | N | ↑ | - | N-↑ | ↑ | ↑ | N-↓ | ↑ | N-↑liver enzymes |
| Grimaud  2020 | Neutrophilia, lymphopenia | N-↓ | N-↓ | ↑ | - | ↑ | - | - | N-↓ | - | ↑fibrinogen |
| Toubiana  2020 | ↑  Neutrophilia, lymphopenia | N-↓ | N-↓ | ↑ | - | ↑ |  | ↑ | ↓ | ↑ | hypoNa.  N-↑creatinine, N-↑liver enzymes |
| Whittaker  2020 | ↑  Neutrophilia, lymphopenia | N-↓ | N-↓ | ↑ | - | - | ↑ | ↑ | ↓ | - | ↑LDH,  N-↑liver enzymes, ↑fibrinogen |
| Blondiaux 2000 | ↑  Lymphopenia | - | - | ↑ | - | - | - | - | - | - | - |
| Cheung  2020 | N-↑  bands, lymphopenia | N-↓ | N-↓ | ↑ | - | ↑ | N-↑ | ↑ | - | ↑ | hypoNa,  N-↑liver enzymes,  N-↑LDH,  ↑PT, aPTT  ↑IL-2R, ↑IL-18, ↑CXCL-9 |
| Ramcharan  2020 | - | - | - | ↑ | ↑ | ↑ | ↑ | ↑ | - | - | ↑CK peak |
| Pouletty 2020 | Neutrophilia, lymphopenia | - | N-↓ | ↑ | - | ↑ | N-↑ | - | N-↓ | ↑ | N-↑creatinine, N-↑liver enzymes |
| Kaushik 2020 | N-↑, lymphopenia | N | N | ↑ | ↑ | ↑ | ↑ | ↑ | N-↓ | - | ↑fibrinogen, N-↑liver enzymes, |
| Greene 2020 | ↑  Left-shifted, lymphopenia | - | N | ↑ | - | ↑ | ↑ | ↑ | - | ↑ | ↑fibrinogen, ↑PT, ↑INR, ↑creatinine |
| Dufort 2020 | N-↑, lymphopenia | - | N-↓ | ↑ | N-↑ | N-↑ | N-↑ | ↑ | N-↓ | ↑ | N-↑fibrinogen N-↑LDH |
| Feldstein 2020 | ↑  Neutrophilia, lymphopenia | N-↓ | N-↓ | ↑ | ↑ | - | N-↑ | N-↑ | N-↓ | - | N-↑liver enzymes, N-↑fibrinogen, N-↑INR |

aPTT: activated partial thromboplastin time; BNP: brain natriuretic peptide; CK: creatinine kinase; CRP: C-reactive protein; CXCL-9: chemokine ligand 9; ESR: erythrocyte sedimentation rate; HyperK: hyperkalemia; HypoNa: hyponatremia; IL: interleukin; INR: international normalized ratio; Ig: immunoglobulin; LDH: lactic dehydrogenase; N: normal; Neg: negative; PCT: procalcitonin; Plts: platelets; Pos: positive; PT: prothrombin time; RBC: red cell blood count; RT-PCR: reverse transcription polymerase chain reaction; TG: triglycerides; TNF: tumor necrosis factor; WBC: White cell blood count

**Supplemental Methods**

*Data Sources and Strategy*

- **Data Sources:** MEDLINE, PMC and NCBI Bookshelf throughout PubMed resource, from inception to June 16^th^, 2020.
- **Search strategy:** ((Covid-19 OR Sars-CoV-2 OR coronavirus-19 OR COVID*) AND (child OR children OR pediatric* OR paediatric*) AND ("multisystem inflammatory syndrome" OR "multi-system inflammatory syndrome" OR kawasaki OR hyperinflammatory OR hyperinflammation)). **Filters:** no filters.
- Additionally, the reference lists of the most relevant identified articles were also searched by hand with the aim of optimizing the searching results.

*Study selection*

- Articles were considered eligible for inclusion if they presented epidemiologic data on MIS-C: case report, case series, observational retrospective and case-control study, observational prospective studies.
- Editorial, comments, letters and review which did not include new epidemiological data were excluded from the case-series review, but they may have been included as literature reference if judged to be relevant to the discussed topic.
